# Case Report: Compound heterozygous variants in *LSS* and *TSPEAR* genes causing hypotrichosis type 14 complicated with ectodermal dysplasia type 14

**DOI:** 10.3389/fmed.2026.1669753

**Published:** 2026-01-20

**Authors:** Yonglong Xu, Dingquan Yang, Ying Xie, Qingwu Liu, Shuying Lv, Meijiao Du, Lei Wang

**Affiliations:** 1 Beijing University of Chinese Medicine, Beijing, China; 2 Department of Dermatology, The National Center for the Integration of Traditional Chinese and Western Medicine, China-Japan Friendship Hospital, Beijing, China

**Keywords:** autosomal recessive, case report, congenital alopecia, LSS, TSPEAR, variant

## Abstract

**Objective:**

To describe the clinical features and genetic findings in a child with hypotrichosis type 14 (HYPT14, OMIM: 618275) complicated by ectodermal dysplasia type 14 (ED14, OMIM: 618180) harboring compound heterozygous variants in *LSS* (OMIM: 600909) and a heterozygous variant in *TSPEAR* (OMIM: 612920), to summarize the potential phenotypic impact of concurrent variants in both genes, and to provide evidence relevant to diagnosis and mechanistic investigation of related diseases.

**Methods:**

Clinical data were collected. Peripheral blood samples from the child and his mother were obtained for next-generation sequencing-based variant screening. Sanger sequencing was used for segregation analysis of candidate variants. In addition, relevant studies were reviewed to contextualize the reported phenotypes associated with *LSS* and *TSPEAR* variants and to discuss potential biological interactions.

**Results:**

The child carried compound heterozygous variants in *LSS*: c.1025T>G; p.(Ile342Ser) and c.3G>A (maternally inherited), and a heterozygous variant in *TSPEAR*, c.872G>A; p.(Arg291Gln). All variants were classified as variants of uncertain significance (VUS) under current ACMG criteria, and a definitive causal relationship with the phenotype cannot be established at present. Nevertheless, the patient’s phenotype showed overlap with reported features of HYPT14 and ED14 and adds clinical data that may support future variant reclassification as additional evidence accumulates. Immunomodulatory therapy, including a JAK inhibitor, produced only a transient response and did not result in sustained hair regrowth.

**Conclusion:**

Coexisting *LSS* and *TSPEAR* variants may contribute to a phenotype of HYPT14 complicated by ED14 in this child. Because the variants remain VUS, genotype–phenotype inferences should be made cautiously. The findings raise the possibility that oligogenic effects may exacerbate ectodermal abnormalities. To our knowledge, this is the first reported case of digenic inheritance involving *LSS* and *TSPEAR*, which expands the clinical spectrum of *LSS*- and *TSPEAR*-associated disorders and supports consideration of broader genetic testing in children with congenital hypotrichosis and ectodermal features.

## 1 Introduction

Hypotrichosis type 14 (HYPT14, OMIM: 618275) is a rare autosomal recessive disorder caused by biallelic variants in the *LSS* (OMIM: 600909) gene. *LSS* encodes lanosterol synthase, a key enzyme in the cholesterol biosynthesis pathway, and loss of *LSS* function leads to abnormal hair follicle development, characterized by congenital total body hair loss or extreme sparseness, accompanied by soft, fine, and easily falling hair (1). Recent studies have revealed significant heterogeneity in the *LSS* variant spectrum; in addition to isolated hypotrichosis, it can also involve the eyes, nervous system, and skin appendages, presenting with syndromic phenotypes such as congenital cataracts, intellectual disability, or palmoplantar keratoderma (2). Ectodermal dysplasia type 14 (ED14, OMIM: 618180) is associated with variants in the *TSPEAR* (OMIM: 612920) gene, which encodes a protein involved in ectodermal tissue morphogenesis. Variants in this gene can result in typical phenotypes, including sparse hair, dental agenesis or deformities, and sweat gland dysfunction (3–5). Notably, due to overlapping regulatory networks in ectodermal development, *LSS* and *TSPEAR* variants may act synergistically to impair hair follicle development. To date, clinical evidence supporting pathogenicity attributable to variants in both genes has not been reported.

This article reports the case of a 7-year-old boy who presented at birth with congenital total body hair loss, nail plate dystrophy, and refractory eczematous dermatitis. Genetic testing identified compound heterozygous variants in *LSS* and a heterozygous variant in *TSPEAR*, fulfilling the molecular diagnostic criteria for HYPT14 complicated by ED14. To our knowledge, this is the first reported case of concomitant *LSS* and *TSPEAR* variants.

## 2 Clinical data

**Figure 1:**
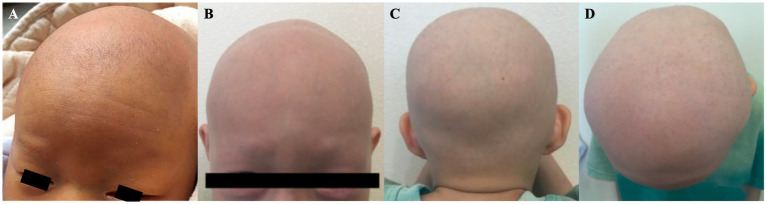
**(A)** Total body hair loss present at birth. **(B,C)** Images obtained at the initial visit showing near-complete absence of scalp hair, eyebrows, and eyelashes.

On August 20, 2023, a 7-year-old boy presented to our hospital. The patient was born at full term by spontaneous vaginal delivery after an uncomplicated pregnancy. Generalized hair loss was present from birth (Figure 1). Over the subsequent 7 years, sparse, fine hair intermittently grew over the vertex of the scalp, but it shed spontaneously after reaching a maximum length of approximately 1 cm. These episodes recurred. No standardized therapy was provided during this period. The patient was otherwise healthy and had no history of in utero drug exposure. He was an only child. His parents were healthy with normal hair growth, were non-consanguineous, and no similar cases were reported in the family.

### 2.1 Physical examination

**Figure 2:**
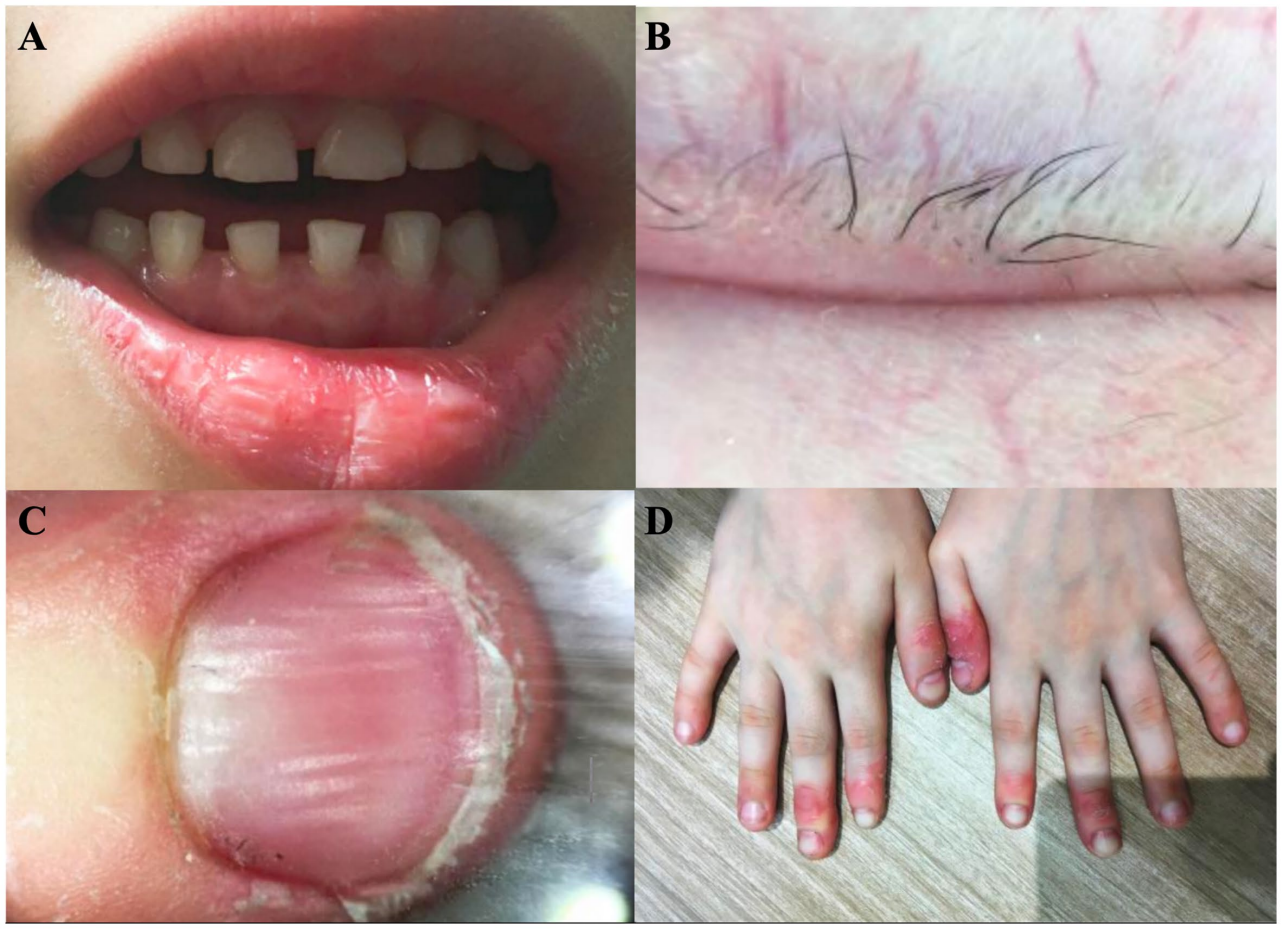
**(A)** Primary dentition showing sparse, hypoplastic deciduous teeth with a trapezoidal shape. **(B)** Eyelid margin showing eyelash loss with visible telangiectasia. **(C)** Nail dystrophy with longitudinal ridging and surface roughness. **(D)** Hand dermatitis showing erythema, scaling, and fissuring of the fingers.

The patient’s height was 115 cm and his weight was 21.5 kg. The primary teeth were sparse and hypoplastic, with a trapezoidal morphology (Figure 2A). Growth and intellectual development were within normal ranges. There was no history of seizures, skeletal deformities, neurological symptoms, or autoimmune diseases. Audiological and ophthalmological examinations were normal, with no evidence of congenital cataracts. Sweating was normal.

### 2.2 Dermatological examination

The dermographism (scratch) test was positive. Scalp hair, eyebrows, and eyelashes were almost completely absent, and axillary and pubic hair had not developed. The skin in the alopecic areas was smooth, without scarring, erosion, inflammation, or scaling. Eyelash loss was accompanied by visible telangiectasia (Figure 2B). The nail plates of the hands and feet were involved, showing surface roughness, multiple longitudinal ridges, and increased fragility (Figure 2C). Erythema, scaling, and fissuring were present on the fingers bilaterally (Figure 2D). The patient had a history of eczema, with an Eczema Area and Severity Index (EASI) score of 1.2 and a Children’s Dermatology Life Quality Index (CDLQI) score of 13.

### 2.3 Trichoscopic examination

**Figure 3:**
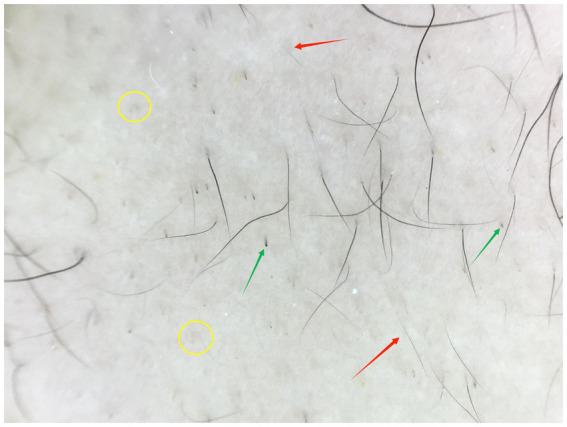
Trichoscopy of the vertex scalp showing broken hairs (green arrows), short vellus hairs (red arrows, length <1 cm), and black dots (yellow circles) with reduced follicular openings (Hongxin, polarized light, ×20).

Trichoscopy showed a decreased density of follicular openings. Broken hairs, black dots, and scattered vellus hairs (length <1 cm) were observed in the alopecic areas (Figure 3).

### 2.4 Blood examination

Complete blood count, hepatic and renal function, thyroid function, whole-blood trace elements, serum total immunoglobulin E (IgE), serum cholinesterase (CHE), tuberculosis antibody (Tb-Ab), hepatitis B serologic markers, and hepatitis C antibody were all within normal limits.

### 2.5 Allergen testing

Food-specific IgG testing showed positivity to eggs, milk, and rice.

### 2.6 Treatment

Based on the initial clinical presentation and trichoscopic findings, which revealed broken hairs, black dot, and short vellus hairs that were highly suggestive of alopecia totalis, the patient was provisionally diagnosed with severe childhood alopecia areata and treated accordingly with compound glycyrrhizin tablets (50 mg once daily), tofacitinib citrate tablets (5 mg twice daily), cyproheptadine hydrochloride tablets (2 mg nightly), and 5% topical minoxidil solution (1 mL once daily). After 5 weeks of medication, thicker and denser hairs appeared on the scalp, with an average length >1 cm, and the Severity of Alopecia Tool (SALT) score decreased to 40%. No obvious adverse reactions were observed, and the therapeutic effect was notable. This initial response was consistent with reported outcomes of JAK inhibitor therapy in refractory pediatric alopecia areata and further supported the working diagnosis at that time. However, hair loss recurred after 6 weeks of treatment. At follow-up examination 7 weeks later (October 7, 2023), hair loss had returned to the initial level with no significant improvement. The treatment regimen was then adjusted to compound glycyrrhizin tablets (50 mg twice daily), with 0.05% clobetasol propionate compound ointment applied topically to the alopecic areas every night, while other medications remained unchanged. Regular follow-ups every 3 months showed no significant improvement by the January 1, 2024 visit. Because the patient’s family initially declined genetic testing, treatment for severe childhood alopecia areata was continued under close clinical and laboratory monitoring, in line with contemporary therapeutic approaches for refractory pediatric alopecia areata. Our team previously observed significant hair regrowth (close to normal) in a child with refractory severe alopecia areata after 6 months of baricitinib treatment (6). Based on this experience and the presumed autoimmune etiology at that time, the regimen was adjusted to oral baricitinib (4 mg once daily) combined with topical minoxidil tincture, and other drugs were discontinued. However, after 6 months of treatment, satisfactory hair growth was still not observed. Trichoscopy showed only sparse hair follicle openings and a small amount of vellus hairs. After genetic testing confirmed compound heterozygous variants in *LSS* and a variant in *TSPEAR*, indicating hereditary hypotrichosis with ectodermal dysplasia rather than autoimmune alopecia, immunomodulatory therapies such as tofacitinib and glycyrrhizin were promptly discontinued because these agents are not expected to be effective for hereditary alopecia. Throughout the entire treatment course, regular monitoring of blood counts and liver and kidney function revealed no drug-related adverse reactions.

## 3 Genetic testing methods and results

**Figure 4:**
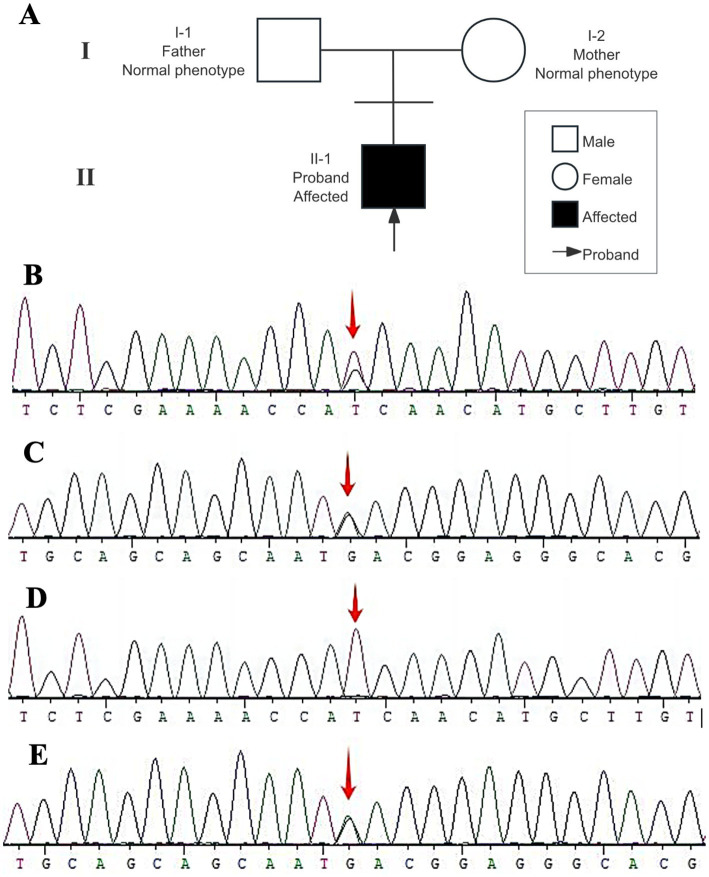
Pedigree and *LSS* Sanger sequencing. **(A)** Pedigree of the family. Squares indicate males and circles indicate females. Filled symbols denote affected individuals and open symbols denote unaffected individuals. The arrow indicates the proband (II-1). Both parents show a normal phenotype. **(B)** Proband, heterozygous *LSS* c.1025T>G p.(Ile342Ser). **(C)** Proband, heterozygous *LSS* c.3G>A (initiation codon). **(D)** Mother, wild-type at *LSS* c.1025 (no c.1025T>G detected). **(E)** Mother, heterozygous *LSS* c.3G>A, supporting maternal inheritance.

A labeled pedigree was constructed based on the available family information to illustrate the familial relationships and the inheritance pattern of the proband (Figure 4A). Following the clinical evaluation and treatment course, genetic analysis was performed to further clarify the underlying etiology. Peripheral blood samples were collected from the patient and his mother for sequencing, and subsequent molecular testing was carried out as described below.

### 3.1 Methods

After obtaining informed consent, peripheral blood samples were collected from the patient and his mother. Genomic DNA was fragmented and used for library construction. Targeted capture was then performed to enrich the coding regions of selected genes and their flanking splice sites. Variant detection was subsequently performed using next-generation sequencing.

### 3.2 Results

Genetic testing revealed compound heterozygous variants in *LSS* (Figures 4B,C): c.1025T>G; p.(Ile342Ser) located in exon 10 (NM_002340.6), which substitutes isoleucine with serine, and c.3G>A in exon 1 (NM_002340.6), an initiation codon variant. Both variants were classified as variants of uncertain significance (VUS). However, these variants have previously been reported in association with HYPT14 and alopecia-intellectual disability syndrome type 4 (OMIM: 618840). Family segregation analysis confirmed that c.3G>A was inherited from the mother, who is a heterozygous carrier (Figures 4D,E). The parental origin of c.1025T>G could not be determined because a paternal sample was unavailable.

In addition, a heterozygous variant in *TSPEAR*, c.872G>A; p.(Arg291Gln), was detected in exon 6 (NM_144991.3). This variant was classified as a VUS and may be associated with ED14 of the hair–teeth type.

## 4 Discussion

This study reports what is, to our knowledge, the first case of HYPT14 complicated by ED14 in which compound heterozygous variants in *LSS* coexist with a heterozygous variant in *TSPEAR*. While all identified variants are currently classified as VUS, the detailed clinical characterization of this patient provides important human phenotypic evidence supporting a potential contributory role of these variants in ectodermal development. The patient’s phenotype encompasses the typical features of both conditions and appears more severe than that reported in previous cases involving single-gene variants. This finding expands the phenotypic spectrum of hereditary hypotrichosis and provides additional context for investigating the molecular mechanisms underlying ectodermal developmental disorders.

The *LSS* gene encodes lanosterol synthase, a key enzyme in cholesterol biosynthesis. The c.3G>A initiation codon variant identified in this study has been reported to abolish protein translation, whereas the c.1025T>G; p.(Ile342Ser), variant is located in a highly conserved catalytic domain and may disrupt the spatial conformation of the enzyme’s active site (7). The resulting impairment of cholesterol synthesis may affect follicular keratinocyte differentiation and hair shaft keratinization, which is consistent with trichoscopic findings of follicular miniaturization and black dots in this patient. However, functional confirmation of these effects is still lacking, and the proposed mechanism should be regarded as putative. The heterozygous *TSPEAR* variant c.872G>A; p.(Arg291Gln), is located in an epidermal growth factor-like repeat domain that is important for extracellular matrix assembly (3). Although this variant remains a VUS, its co-occurrence with ectodermal developmental abnormalities such as dental hypoplasia and nail dystrophy suggests that it may modify ectodermal development by perturbing relevant signaling pathways. Notably, a functional interaction between *LSS*-mediated cholesterol metabolism and *TSPEAR*-regulated extracellular matrix signaling is plausible. Cholesterol influences membrane organization and can affect epidermal growth factor receptor localization and activation. Altered *TSPEAR* function may reduce extracellular matrix support for signaling components, potentially amplifying the phenotypic impact of impaired cholesterol synthesis. This interaction may contribute to the increased severity observed in this case relative to reports involving single-gene variants.

This case exhibits significant phenotypic differences compared to the case reported by Yang et al. (7), which carried the same compound heterozygous *LSS* variants. The patient reported by Peled et al. (8) presented with palmoplantar hyperkeratosis, corpus callosum hypoplasia, and early-onset cataracts. In contrast, our patient primarily manifested total body hair loss, dental hypoplasia, and nail dystrophy, without skin keratinization, neurologic abnormalities, or ocular involvement. Such differences may reflect multiple interaction factors, including the additional heterozygous *TSPEAR* variant, which may influence dental germ development and potentially modulate cutaneous keratinization pathways. They may also reflect inter-individual variation in the expression of truncated proteins, leading to differences in the degree of cholesterol synthesis impairment (5). In addition, long-term eczematous dermatitis and topical glucocorticoid exposure might have partially masked keratotic features. Together, these observations indicate that identical variants can be associated with phenotypic divergence due to genetic background and environmental influences, which complicates simple genotype–phenotype correspondence. They also suggest that palmoplantar keratoderma–congenital alopecia syndrome type 2 may represent a phenotypic subtype within the *LSS*-related spectrum rather than an independent entity. Accordingly, we suggest extended phenotypic evaluations for patients with pathogenic or likely pathogenic *LSS* variants, including cranial MRI, ophthalmic examinations, and dental assessments. For patients with atypical phenotypes, additional screening of ectodermal development genes such as *TSPEAR* may help identify modifying factors.

To date, no cases of *LSS*-related disorders with concurrent *TSPEAR* variants have been reported. As the first molecularly characterized case of ectodermal dysplasia with coexisting *LSS* and *TSPEAR* variants, this report challenges a strictly single-gene framework and raises the possibility that oligogenic effects may contribute to phenotypic overlap and increased severity in rare diseases. Nevertheless, given the current VUS status of these variants, this concept remains hypothesis-generating and requires validation in further clinical and experimental studies. This case also provides clinical support for potential crosstalk between cholesterol metabolism and extracellular matrix signaling in hair follicle development. It may inform diagnostic workflows by supporting dental assessment and consideration of *TSPEAR* testing in patients with HYPT14 to facilitate earlier recognition and counseling.

This study has several limitations. First, the *LSS* and *TSPEAR* variants are currently classified as VUS, and a definitive genotype–phenotype causal relationship cannot yet be established. The primary value of this report lies in providing detailed phenotypic data that may support future variant reclassification as evidence accumulates. Second, due to the unavailability of the father’s sample, the origin of the c.1025T>G variant in the *LSS* gene cannot be clarified. Third, functional validation of the *TSPEAR* variant has not been performed, and its pathogenic relevance requires further investigation. In addition, long-term phenotypic evolution remains to be defined. Future studies could investigate the interaction between these genes using organoid models or transgenic systems, thereby laying the foundation for exploring targeted therapeutic strategies.

## Data Availability

The original contributions presented in the study are included in the article/supplementary material, further inquiries can be directed to the corresponding authors.
